# The Nutrition-COVID-19 Interplay: a Review

**DOI:** 10.1007/s13668-021-00380-2

**Published:** 2021-11-27

**Authors:** Janet Antwi, Bernard Appiah, Busayo Oluwakuse, Brenda A. Z. Abu

**Affiliations:** 1 Department of Agriculture, Nutrition and Human Ecology, Prairie View A&M University, Prairie View, TX USA; 2 Department of Public Health, Syracuse University, Syracuse, NY USA; 3 Wegmans School of Health and Nutrition, College of Health Sciences and Technology, Rochester Institute of Technology, Rochester, NY USA

**Keywords:** Nutrition, COVID-19, Immune system, Double burden of malnutrition, Nutrition behavior, Weight changes, Food insecurity, Ageusia, Dysgeusia, Lockdown

## Abstract

**Purpose of Review:**

Nutritional status is affected by the COVID-19 pandemic, directly or indirectly. Even with the recent rollout of the coronavirus disease 2019 (COVID-19) vaccines and availability of medicines such as remdesivir, and monoclonal antibodies, host nutritional status is pivotal in the fight against the acute respiratory syndrome coronavirus 2 (SARS-CoV-2) infection and outcomes. The purpose of this review is to discuss the effects of COVID-19-related lockdown on lifestyle behaviors, and the nutritional consequences, and the direct sequelae of the infection on nutrition including potential nutritional interventions.

**Recent Findings:**

The COVID-19-related lockdown imposed radical changes in lifestyle behaviors with considerable short-term and long-term health and nutritional consequences including weight gain and obesity and increased cardiometabolic risk, consistently linked to worsened prognosis. The extent of the impact was dependent on food insecurity, overall stress and disordered eating, physical inactivity, and exposure to COVID-19-related nutrition information sources. COVID-19 could directly induce inflammatory responses and poor nutrient intake and absorption leading to undernutrition with micronutrient deficiencies, which impairs immune system function with subsequent amplified risk of infection and disease severity. Nutrition interventions through nutrition support, dietary supplementation, and home remedies such as use of zinc, selenium, vitamin D, and omega-3 fatty acids showed the most significant promise to mitigate the course of COVID-19 infection and improve survival rates.

**Summary:**

The nutrition-COVID-19 relationship and related dietary changes mimic a vicious cycle of the double burden of malnutrition, both obesity and undernutrition with micronutrient deficiencies, which promote infection, disease progression, and potential death.

## Introduction

Coronavirus disease of 2019 (COVID-19), which is described as severe acute respiratory syndrome caused by the newly identified coronavirus 2 (SARS-CoV-2), has been recognized by the World Health Organization in the past year, 2020, as a deadly pandemic [1]. Its effect on psychosocial and physiological processes and rise in cases are still being dealt with despite ease of lockdown and ramp-up in vaccinations. As the scientific and intelligence communities warn of the devastating long-term effect of the COVID-19 pandemic, there is yet to be an approved specific pharmacological treatment, making boosting the immune system crucial [2, 3, 4••, 5]. The COVID-19 pandemic has directly and indirectly caused an observed increase in the double burden of malnutrition, a phenomenon that previously pertained mainly to developing countries, to be experienced worldwide [6–10]. The co-existence of undernutrition with micronutrient deficiencies notwithstanding, the increasing prevalence of overweight/obesity and diet-related chronic diseases, has been associated with the COVID-19 infection itself, and its collateral damages. These include induced loss of sense of smell and taste, food insecurity, and COVID-19-related lockdown on lifestyle behaviors of excessive intake of unhealthy food and physical inactivity [11•, 12••, 13, 14••]. Malnutrition in all its forms, particularly undernutrition and obesity, alters the immune response which is a driving force for the prevention, treatment, and progression of COVID-19.

The experience with COVID-19-related quarantine has caused changes in dietary habits and lifestyle parameters, which may lead to impaired nutritional status. The COVID-19 pandemic led to lockdowns, which has impacted availability, accessibility, and affordability of safe nutritious food leading to food insecurity, inadequate, or unhealthy food intake among some individuals and households [13]. This makes food insecurity a “double-edged sword,” causing both undernutrition and overnutrition [15–18]. COVID-19 exacerbated weight gain through food insecurity, a primary driver of nutrition behavior, due to continued lack of access to adequate safe nutrient-rich foods. The closure of global borders to food trade, shortfall in food production, and loss or decline in household income during the COVID-19 pandemic lockdown caused significant shifts in food availability necessitating a resort to unhealthy food choices [19–21]. It is believed that increased stress and anxiety from fear of contracting the disease and social isolation increased emotional eating characterized by excessive intake of calorie-dense foods [22]. Such negative nutritional behaviors reported during this period include increased consumption of foods high in saturated fat and sodium and with added sugars, late night eating. In addition to increased physical inactivity, these negative behaviors lead to excessive weight gain, thereby increase obesity, a major risk factor associated with high vulnerability to the viral infection, COVID-19 progression, and mortality [11•, 12••]. Obesity is a state of chronic low-grade inflammation which promotes decreased immune response to the coronavirus [23]. Available evidence suggests that the increase in inflammation that generally occurs in the nutritional status of undernutrition also occurs in obesity [24]. This suggests that obese and undernourished patients are more vulnerable to develop the most severe forms of COVID-19. Furthermore, obesity sharply increases the risk of cardiovascular diseases, type 2 diabetes, and hypertension, three of the most serious underlying medical conditions which increase susceptibility and severity of COVID-19 [25].

During infection with the SARS-CoV-2, there is an immune system perturbation generating an immune response, which is key to fighting, eliminating, and preventing virus progression. A weakened host immune system enables the virus to propagate, resulting in aggressive inflammation and extensive tissue damage in organs rich in angiotensin-converting enzyme 2 (ACE2) receptors, a key receptor required by the SARS-CoV-2 to enter host cells [26–28]. The inflammatory response generates appetite loss and modification of intestinal absorption limiting the intake, uptake, and utilization of macronutrients and key micronutrients resulting in undernutrition with micronutrient deficiencies [29–33]. Undernutrition in turn aggravates inflammation regulation, impairs immune function, and increases the risk of infection [26, 27]. Moreover, protein energy-malnutrition and micronutrient deficiencies due to poor nutrient intake may result from COVID-19 induced alterations in taste and smell among those infected [14••].

The nutrition-COVID-19 relationship mimics a vicious cycle of the double burden of malnutrition, both obesity and undernutrition with micronutrient deficiencies, which promote infection, disease progression, and potential death. Many studies and reviews conducted have focused on only how nutrition could modulate COVID-19, but there are few studies and reviews on the impact of COVID-19 on nutrition and lifestyle behavior or discussion of both perspectives. Reviews that simultaneously describe the relationship and implications of nutrition on COVID-19 and vice versa are urgently needed. Such reviews could facilitate the design of timely nutrition interventions to ensure continued adequate nutrient intake, promote health, and quality of life even as the COVID-19 pandemic lingers. This research reviews the literature on the impact of COVID-19 on nutrition to gain a better understanding of this relationship.

## Impact of COVID-19 Lockdowns on Nutrition and Lifestyle Changes

COVID-19 has had a major destructive impact on economic, food, and health systems. To reduce the spread of the virus lockdowns were enforced leading to restricted movements, and changes in work formats and approaches. Consequently, food choices and nutrient intake, physical activity, and lifestyle behaviors were also impacted, leading to both short-term and long-term effects on body weight, and overall health [12••, 34, 35, 36••].

### Nutrition Behaviors and Body Weight

Understanding how COVID-19 impacts people’s dietary behaviors is critical. Various cross-sectional studies have shown marked increase in behaviors that promote obesity during COVID-19, but longitudinal studies are lacking [37, 38]. With changes in body weight, body mass index, and lifestyle behaviors in the Unites States adults during the COVID-19 pandemic, Bhutani et al. [12••] collected and analyzed data during the height of lockdown (March–May 2020) and after (September–October 2020). Results showed that body weight and body mass index significantly increased at the post-lockdown as compared to the peak-lockdown period. About 40% of participants reported to have gained weight of between 1 and 5 lb or greater during the peak-lockdown. The authors further reported that weight gainers frequently consumed ultra-processed foods, snacking, physically exercised less, were in a state of high stress, and could not control their cravings during the peak. Likely cascading effect of weight gain was reported in another study in the Netherlands [35], where overweight and obese individuals were more likely to purchase chips/snacks, and non-alcoholic beverages compared to individuals with healthy body weight. These findings were confirmed in another study [39•]. Similar findings were observed among adults in 28 countries in the Middle East and North Africa (MENA) region [36••]. In Zimbabwe, although 44.5% gained weight, 24.3% lost weight and 31.2% did not have weight change [40].

Short-term and long-term weight gain and changes during the lockdown were mainly modulated by dietary choices during the lockdown. For instance, a study that reviewed different diets in Spain reported negative changes to dietary patterns since lockdown, with COVID diet being high energy-dense, lower in overall nutritional quality, and higher environmental impacts [41]. The paucity of dietary guidelines for emergencies such as a lockdown was documented, indicating the need for further planning and integration of guidelines [41]. Physical and psychological isolation associated with the lockdown and increased reliance on video conferencing during the COVID-19 lockdown led to undesirable coping behaviors such as eating disorder cognitions and attitudinal challenges. Such attitudinal changes in eating behavior resulted in overeating or binge eating to severe caloric restriction [37, 38]. Moreover, the uncertainty surrounding COVID-19 pandemic, the devastating impacts on the infected and affected, and restricted movements introduced stress and anxiety among many people. A study in Italy showed that among 602 respondents, various mental distress in the forms of depressive moods (61.3%), anxiety (70.4%), hypochondria (66.2%), and insomnia (52.2%) were experienced during the lockdown [22]. Di Renzo and co-authors showed an overall increase in the consumption of comfort foods, although specific foods were not listed. Females and younger adults were the most affected. These findings were supported by a study conducted among 5738 French undergraduate students only 7 days after the lockdown was introduced [34].

Stress related to binge eating and restrictive eating were reported. Even though a higher exposure to COVID-related media was a strong determinant, females, low impulse regulation, high body dissatisfaction, and concurrent eating disorder posed the greatest risk of manifesting the two dietary impacts [34]. Fernández-Aranda and co-authors [42] reported that a total of 121 clients with eating disorders reported exacerbated effects on symptomatology and psychopathology because of COVID-related confinement. A newly developed COVID Isolation Eating Scale (CIES) confirmed these findings. Some of these patients expressed dissatisfaction and accommodation difficulty regarding remote therapy when compared with the previously provided in-person therapy, especially for anorexia nervosa [42]. For children, remote learning associated with lockdown reported some stress-inducing activities. A study in Polish primary school students showed that 1334 adolescents aged 10–16 years reported increased screen time [43] likely increasing stress. According to Shen et al. [38], emotional eating mediated the link between perceived stress levels and motived food choices during the pandemic as observed in a study of 800 US adults, with most of the respondents (73.6%) experiencing moderate to high levels of perceived stress, which was significantly linked to emotional eating and more than half of the food choice motives studied.

On the contrary, the lockdown may have had some positive effects on dietary choices. For instance, fast food and ordered foods intake decreased [39•]. Fast food intake is associated with high caloric intake [44], poor diet quality [45], and health outcomes related to increase calories, fat, and sodium [46]. Another positive dietary practice reported during the lockdown was an increase in home meal preparation, as a result of the shutdown of restaurants and bars during the pandemic. Homemade food has been generally more nutritious than food cooked in restaurants, because individuals can make healthier choices during cooking [47]. Another factor that could contribute to this positive effect in dietary choices is the concern of consuming healthier food as a way of boosting immune system against diseases. In China, a study of 10,082 participants aged 15–28 years old found that during the COVID-19 lockdown, there were significant decreases in intake of foods such as rice, meat, fresh vegetables, fresh fruit, soybean products, and dairy products, but significant increases in the intake of wheat products and preserved vegetables [48]. In Poland, a study among 1016 children and adolescents aged 6–15 years identified reduced intake of foods such as fruit juices, sweetened drinks, diet sodas, meats and canned food, fast food, and snacks but increased consumption of dairy products, fish, poultry, and meat during the lockdown [49]. A similar study in Poland showing improved nutritional behaviors among 1334 adolescents aged 10–16 years old was found [43].

### Food Insecurity and Dietary Changes

The year 2020 to date has made a huge mark in the increase of the condition of global food insecurity. Food insecurity can be defined as an insufficient or uncertain access to nutritious food for a healthy well-being [50]. Food insecurity and poor nutrition are associated with underweight with micronutrient deficiencies and obesity and chronic illness complications, and as such, people are placed at risk of COVID-19 virus [20, 51–53]. Food insecurity was a natural consequence of the lockdown [54]. COVID-related deaths of household heads, job losses and transition, pay cuts, food shortages, and restricted scope of food assistance programs impacted food access to many, especially for individuals from low-income households [54, 55]. For instance, since the pandemic began, many surveys in the United States have documented high rates of food insecurity that exceeds that identified in the recent decades. The United States Department of Agriculture estimated food insecurity in America to be around 11 to 12% in the last 5 years [50]. From March to April 2020, the national estimates of food insecurity increased three times to about 38%. Within the United States in March 2020, food insecurity was identified in 44% of all households, with 48% of these households being Black households, 52% being Hispanic, and about 54% having children [56]. Households that spend most of their incomes on food were affected the most. In South California, a longitudinal study showed that 40.5% of those residing in households with about $75,000 earnings a year and who were hired in February 2020 lost their job as a result of the pandemic [57]. Thirty-one percent among that group mentioned food insecurity while 33% indicated that they were eating less because of COVID-19-induced inadequate finances. Likewise, in Zimbabwe, marked increase in food prices and decrease in availability of nutritious foods during the lockdown was reported [40]. According to 1075 adult participants from 82 countries including Africans, food prices especially for cereals and legumes directly affected food acquisition (32.7%), dietary diversity (50.4%), and quantity (39.2%) of foods [58]. In addition to increased food prices, food waste within the food system related to the disruption [59] and at the household level [60] impacted food access. Vulnerable individuals and households are the most at risk of food insecurity.

Online surveys of the United States Supplemental Nutritional Assistance Program (SNAP) indicated that household food insecurity and financial debt exacerbated significantly between April and June of 2020 [50, 61]. The pandemic has also began affecting food security in households that already suffered battling debt, job losses, or life-threatening illness before the pandemic began. Coping strategies of new or consistent households experiencing food insecurity involved disrupted eating patterns also known as consuming less food, buying cheaper foods which are nutritionally inadequate, and relying on food handouts from friends, family, and government-assisted programs. In comparison to the food from secure households, households that fell into the less secure ones were significantly more likely to receive assistance pay bills, and tended to rely heavily on existing food assistance programs [13, 21]. Despite various financial and nutrition assistance programs including the Families First Coronavirus Response Act 4 and the Coronavirus Aid, Relief, and Economic Security (CARES) Act 5, emergency SNAP allotments and extensions, delivery of food to homes, and boosting the SNAP benefit to the maximum allowable amount to buffer rising food insecurity during this pandemic, not all households who need it have received it, the maximum SNAP benefit has not been increased which means about 40% of SNAP recipients with lowest incomes did not receive additional assistance, and the stimulus check reliefs expired [62, 63]. With the COVID-19 pandemic lingering and expected long-term impact, there is the need for real-time monitoring and data collection to better inform public health experts and federal agencies on policy decisions of individuals and households who need aid and assistance and complement existing safety nets. Unfortunately, most low-income countries lack social network foods compounding food insecurity.

### Physical Inactivity and Lifestyle Changes

While the COVID-19 restrictions during lockdown helped with reducing the spread of the virus, it has resulted in negative effects by limiting daily physical activities that people were used to like travelling and some forms of exercising. Restricted movements and related changes in work formats contributed to major changes in physical activity levels. People generally replaced outdoor activities with sedentary behaviors [54]. In an international online survey that included participants from the United States, Ammar et al. [11•] investigated the behavioral and lifestyle consequences of COVID-19 restrictions on the population. The researchers reported that there was a decrease in all physical activity intensity levels and daily sedentary time increased from 5 to 8 h per day. A third of adults in the United Arab Emirates reported that they did not engage in any physical activities during the lockdown (38.5%), and 36.2% spent over 5 h on screens for entertainments [36••]. Increased screen time has been associated with snacking alongside thereby increasing snacking rate, such as fast foods and soda drinks which leads to overeating [64]. Screen time spent on online grocery shopping, television watching, and playing of video games were the commonly documented [54]. The closure of gyms and exercise centers further impacted individuals who were physically active pre-lockdown. These findings are unfortunate since increase in physical fitness serves as positive correlation with boosted immune function, reduced systemic inflammation, and the ability to cope with infections and the immunologic cardiopulmonary complications. Physical inactivity around the globe is an important predisposition factor for morbidity. It must be noted that the elderly and chronologically ill patients are at greater risk of COVID-19 morbidity [64]. Other lifestyle changes included sleep disturbances [36••]. Sleep disturbances were reported among 60.8% of the same population [36••]. Additional to reduction in physical activity levels, alcohol and smoking behaviors increased among Zimbabwean adults (31–40 years) [40].

### Communication and Nutrition Information Sources

A positive development was reported; however, globally, immune-boosting nutrients and herbs and related knowledge seemed to be the most prioritized regarding food choices. These foods saw the most searches in Google, many seeking information on both natural home remedies and supplements [54]. Turmeric, garlic, and ginger were the commonest remedies, while vitamins A, C, E, and D were the most searched immune boosters [54]. These foods and nutrients have merits for health; however, the focus on overall health was ignored. Similarly, a study conducted by Masters [65] found that people did frantic searches for information and were overly concerned about immune-boosting interventions and food security.

During the lockdown, the search for nutrition-related information outside of Google has been explored by some studies. For example, a study in the United Arab Emirates [66] found that the most cited source of nutrition-related information was social media (67.8%), followed by local and international health authorities (48.7%), healthcare professionals (45.7%), friends and family (38.1%), television (17.0%), and newspapers (5.0%). Similarly, a study involving 18 countries in the Middle East and North African region identified most respondents (70.8) citing social media as source of nutrition information during the lockdown compared with 3.7% for newspapers and 41.3% for healthcare professionals [36••]. These studies suggest the need for nutritionists and other healthcare professionals to consider using social media to implement interventions for improving healthy nutritional behaviors.

The use of technology as a potential solution to continue nutritional services and reduce the impact of the virus and lockdown among individuals with pre-existing conditions has been explored. In a randomized controlled trial, telemedicine was used as a tool for dietary intervention among 55 non-alcoholic fatty liver disease-human immunodeficiency viruses (NAFLD-HIV) patients during the COVID-19 lockdown [67]. In 3 months, the intervention group received follow-up consultations using video and/or phone to promote the Mediterranean diet and the control received general dietary recommendations. Although both groups gained weight, the control group were more likely to report dissatisfaction with their dietary intake during the lockdown or increased appetite compared to the intervention group. The control group showed significant increase in the blood glucose levels [67]. Three supervised interventions (grouped as an educational intervention on healthy habits, a nutritional intervention, and a teleconnection aerobic and strength exercise using intervention) implemented in Spain for 18 weeks were also tested in a randomized control trial among 23 middle-aged university employees [68]. Health-related quality of life was higher especially in areas related to health responsibility, physical activity, and nutrition among the intervention group compared to the control. The intervention reduced sitting time by 2.5 h in a day [68]. The maintenance of healthy dietary and lifestyle behaviors during the pandemic is crucial in the fight against the virus and essential for mental health and well-being. Limited access to fresh food and physical inactivity could negatively affect the overall physical state of an individual worsening an infection.

## COVID-19 Induced Undernutrition with Micronutrient Deficiencies and Nutrition Therapy

Nutritional status can be negatively affected by viruses including the SARS-CoV-2 itself and its related consequences. COVID-19 impacts on the immune response, modulation of inflammatory pathways, resulting in undernutrition with micronutrient deficiencies which can further slowdown recovery. While the literature pertaining to COVID-19 and undernutrition with micronutrient deficiencies are still limited, however, the state of inflammatory response, loss of smell and taste, decreased appetite and reduced food intake, and impaired nutrient utilization are indications for adequate nutritional support and repletion.

### Loss or Blunting of Taste and Smell

Anosmia/hyposmia, the complete or partial loss of smell, and dysgeusia/ageusia which is the partial or complete loss of taste have been identified as highly prevalent symptoms of COVID-19 [69–71]. In several self-reporting surveys, the prevalent rates of smell and taste impairment ranged from 11 to 87% in among those with severe and mild-moderate cases, respectively, and could persist from 7 days to several weeks, with these figures underestimating the true prevalent rates [72]. It is unclear how COVID-19 impairs smell and taste, but it is being hypothesized that the SARS-CoV-2 targets the ACE2 receptors that are found in abundance in the olfactory and gustative systems to invade the nerve cells associated with smell and taste senses [73]. Another possible explanation is that the infection through the nasal passage causes high levels of inflammation in the cells, a potential damage of the ability to smell [74, 75].

Sense of smell and taste are strongly connected; thus, a person’s loss of sense of taste is observed as a resulting factor of loss of smell. The senses of smell and taste play a key role in food choices and food intake. Because they are intricately linked, one or both can affect the eating experience. Although these sensory properties affect food choice, there is limited evidence supporting the link between smell and taste function and nutritional status. Some consequences of changes in smell and taste range from decreased appetite, reduced enjoyment of food, and loss of interest in food [76–81]. For example, some people who experience the loss of sense of smell may lose the ability to taste different flavors but might still be able to distinguish the differences between tastes of sweet, salty, bitter, sour, and umami. As appetite lessens and the eating experience becomes less enjoyable, nutritional intake consequently decreases, thereby putting one at a higher risk of malnutrition. Considering the important role that smell and taste play in eating behaviors, the anosmia/hyposmia and dysgeusia/ageusia experienced during COVID-19 may contribute to inadequate intake causing undernutrition with micronutrient deficiencies, and undesired weight loss. Therefore, it is necessary to ensure that a person continues to feed well during COVID-19 infection to boost their immune system. One strategy is to provide micronutrient supplementation such as zinc which is an appetite stimulant alongside other micronutrients including vitamins D, C, and A and macronutrients in their appropriate proportions. Additionally, it is crucial during this time to provide selective flavor fortification to foods. This requires knowledge in cooking skills and knowledge of nutrition, making nutrition education a crucial intervention during this pandemic, considering most Americans eat out and cook less at home due to lack of culinary and cooking skills.

### Increased Energy and Micronutrient Demand — Nutritional Interventions Targeting COVID-19 Infection Management and Recovery

In the case of an infection, there is an increase in the energy demands and the adequate intake of essentials like carbohydrates, proteins, and lipids must be considered. During critical illness and hospitalization, inadequate nutritional status and malnutrition may prolong length of stay in intensive care units, further leading to muscle mass loss due to reduced mobility, and poor clinical outcomes [82••, 83–88]. The American Society for Parenteral and Enteral Nutrition (ASPEN) guidelines recommend that malnutrition should be considered as part of the management of COVID-19 patients to improve outcomes [89••]. Given the GI effects of COVID-19, a fraction of infected patients have poor appetite and would not be able to meet their nutrition goals with oral diet alone. For this at-risk group, which includes those who are critically ill, enteral nutrition is the preferred route to promote gut integrity and immune function [90]. Patients who are at nutritional risks should receive nutritional support as soon as possible majorly to increase protein intake by consuming oral nutrition supplements. The ASPEN guidelines also recommend that nutritional intervention and therapy should be considered as an integral part of the approach for the patients. Optimal nutritional care followed with life support therapy could improve recovery from this life-threatening disease [91].

A review of studies conducted during the pandemic showed that adequate intake of zinc, selenium, and vitamin D is key for resistance to viral infections, immune function, and reduced inflammation. Adequate levels of these nutrients or early supplementation especially in high-risk individuals might prevent and mitigate the course of COVID-19 infections [92]. In addition to these nutrients, essential fatty acids, specifically, omega 3 polyunsaturated fatty acids showed similar properties in a double-blinded randomized control trial study on 128 critically ill patients infected with COVID-19 [93]. In this trial, the intervention group (*n* = 42) had a higher 1-month survival rate, and higher levels of arterial pH, bicarbonate, and base excess and lower levels of blood urea nitrogen, creatinine, and potassium compared with the control group (*n* = 86) [93].

Home remedies such as the use of ginger, turmeric, and garlic for the prevention and management of COVID infections have been reported [54]; however, clinical trials substantiating effects have not been published. A randomized control trial protocol investigating the effect of a ginger-based herbal tablet in the recovery rate of clinical symptoms, such as fever, dry cough, tiredness, and gastrointestinal symptoms as well as non-clinical features, such as thrombocytopenia and lymphocytopenia, within a week of randomization has been published [94]. The trial compares the effect of the ginger-based tablet at 1000 mg three times daily in addition to standard treatment regimen with controls only receiving the standard treatment regimen among 84 confirmed COVID patients (≥ 18 years) [94]. The findings will provide important guidelines for both clinical and non-clinical management of COVID-19. More of such trials are urgently needed.

In spite of limited data, some countries have provided additional guidelines to the current ASPEN for infected patients [89••]. These additional guidelines sometime include unique diets, additional nutrition support, and services as especially to patients with pre-existing conditions. For instance, in China, the Society for Inflammatory Bowel Disease provided guidelines on how immunosuppressive agents and biologics and diet should be used in addition to virtual support for infected patients who had pre-existing inflammatory bowel disease [95]. These interventions were to avert possible fatal effects considering the new evidence of possible negative effects of COVID infections on gastrointestinal health and function [90, 96].

## Conclusion

The SARS-CoV-2 and subsequent COVID-19 pandemic is still being researched upon, and more findings are being discovered regarding how this affects human life and what it means for the coming years. COVID-19-related changes in dietary habits and lifestyle constraints may lead to poor nutritional status with coexistence of obesity and related chronicity and underweight/unintended weight loss and micronutrient deficiencies, both physiological alterations which lead to higher susceptibility to COVID-19. The place of nutrition, in the absence of specific therapeutic treatment and even with the availability of vaccines, for an effective functioning of the immune system to aid in the management of the COVID-19 disease cannot be overemphasized. A healthy balanced diet with the appropriate myriad of macronutrients and micronutrients supports disease-reducing immunity.

With new SARS-CoV-2 variants emerging and no end in sight soon to the current pandemic globally, it is crucial to escalate the ongoing clinical trials, nutrimetabolomics, and cell signaling dynamics of several nutrients and bioactive compounds and their link with the immune system in nutrition science research to establish nutrition at the frontlines in fighting COVID-19. Such challenging times present an important opportunity for dietitians, nutritionists, and nutrition scientists to give clear advice and recommendations to the population and the medical community, respectively, on the impact of nutritional status in COVID-19 outcomes. Such advice can be given through culturally appropriate communication avenues including use of the mass media and social media. Teaching Kitchens are learning laboratories which use a holistic and interdisciplinary approach where individuals are given real-world, hands-on guidance to cook, eat, move, and think more healthfully, and this could be promoted to foster individualized or personalized nutrition for greater success. It should be emphasized that public health, health, and federal policy makers must also consider nutritional status in food insecurity or social safety-net policies to diminish the impact of COVID-19.
